# Attitude toward Aging Mediates the Relationship between Personality and Mental Health in Older Adults

**DOI:** 10.3390/healthcare9050594

**Published:** 2021-05-17

**Authors:** Teshome Sirak Bedaso, Buxin Han

**Affiliations:** 1 Institute of Psychology, Chinese Academy of Sciences, Beijing 100101, China; teshomesirak@gmail.com; 2 Department of Psychology, The University of Chinese Academy of Sciences, Beijing 100049, China

**Keywords:** attitude toward aging, depression, life satisfaction, personality, Ethiopian elderly

## Abstract

This study aimed to examine attitude toward aging as a potential mediator of the relationship between personality factors and mental health in terms of depression and life satisfaction among older adults. A cross-sectional study was conducted with 438 Ethiopian elderly individuals aged 60 to 69. The results of the regression-based path analysis showed that after adjusting for demographic data, the relationship between agreeableness and depression in older adults was partially mediated by attitude toward aging. Likewise, attitude toward physical change due to aging and psychological growth subscales jointly mediated the correlation between neuroticism and depression. However, a significant direct path between neuroticism and depression persisted. On the contrary, openness had no significant direct association with depression apart from an indirect through psychosocial loss. The link between life satisfaction and agreeableness as well as openness to experience were partially mediated by psychosocial loss. Therefore, a person’s attitude toward aging and personality characteristics should be taken into consideration while designing interventions for managing mental health issues among older adults.

## 1. Introduction

Many studies concerning the association between personality factors and elderly people’s mental health were yielding mixed results. Some scholars associate the reappearance of depression during later life with neuroticism [1]. Nonetheless, according to Koorevaar et al. [2], the onset of depression is more likely associated with higher level of openness. Many scholars agree that a higher score of neuroticism and a lower score of extraversion are significantly correlated with depression [3,4,5]. Personality traits could also predict older adults’ attitude toward aging [6]. However, studies on the mediating role of attitude toward aging in the relationship between the personality traits and mental health in developing countries are scarce.

### 1.1. Attitude toward Aging and Mental Health

While most research has focused on the attitudes of young people toward the elderly, some studies have examined the attitude of older individuals toward themselves. Nevertheless, it is also essential to investigate how older adults perceive their aging and its relationship with their mental health. While a majority of elderly individuals perceive their aging positively [6], some perceive it negatively. Either way, their daily activities would be affected accordingly.

For instance, having a positive attitude toward aging would be more likely to increase physical activities and social participation [7]. However, the role of age [8], cultural context [9], personality [6,8], and health conditions cannot be underestimated [10].

Notable pieces of evidence from previous studies show that attitudes toward aging play a significant role in mental health. Attitudes toward self-aging reported a correlation with depression [8]. Individuals with positive perceptions of their aging also reported better life satisfaction and psychological well-being [11]. Similarly, positive attitude regarding one’s own aging predicts an increase in satisfaction with social support [12]. Conversely, a more negative perception of aging was found to have positive correlation with lower consciousness of age-related changes [13].

Positive attitudes regarding personal aging are helpful in reducing negative psychological symptoms (depression), which might be linked to age-related changes [14]. However, a number of factors might predict an individual’s attitude towards one’s own aging. Several researchers [8,11] have emphasized on the Big Five personality traits as significant contributors to a person’s views of the aging experience. Further research is required to determine whether attitudes toward aging explain the relationship between the Big Five personality traits and mental health among older adults.

### 1.2. Personality and Mental Health

The relationship between personality and mental health is becoming a popular area of study. Personality is considered to be stable in adulthood [15] with slight changes on the level of some traits—neuroticism amplifies during old age, while extraversion and conscientiousness declines [16]. Personality is an essential factor in subjective well-being [17,18], which generally refers to how individuals evaluate their life satisfaction and experiences, from depression to joy [19].

The relationship between the Big Five personality traits and well-being has been studied previously. Past findings reported that high scores on agreeableness were found to be significantly associated with higher levels of well-being in older adults [18]. In their study, Agteren and Iasiello [20] asserted that well-being and mental health are highly interrelated elements. Besides, recent studies measured depression and life satisfaction as indicators of positive and negative mental health [21,22]. Therefore, the Big Five personality traits are important predictors of mental health, measured in terms of depression and life satisfaction.

Regardless of the direction, recent studies reported a significant correlation between all five personality traits and depression [4,5]. Koorevaar et al. [3] also found that extraversion and agreeableness were negatively related to general depression in later life. Furthermore, agreeableness and openness were found to be negatively associated with depression, while neuroticism correlated with depression [23]. According to Weber et al. [5], openness, agreeableness, and neuroticism were positively related with depression. Moreover, neuroticism and extraversion were reported to have a strong indirect mediating effect on depressive symptoms. Neuroticism also had strong direct and indirect effects through aging perception on depression [4]. However, O’Shea et al. [4] did not include the role of personality on the positive aspect of mental health (life satisfaction).

The Big Five personality types are also important predictors of life satisfaction [24]. Lower levels of neuroticism and higher levels of extraversion were strongly related to life satisfaction [17,25]. In addition, agreeableness and conscientiousness were found to be predictors of life satisfaction among retired individuals [25]. Except for neuroticism, which was negatively correlated, the other four personality factors were significantly and positively correlated with life satisfaction [26]. Furthermore, a recent finding suggests that cultural difference is a key factor in the correlation between life satisfaction and neuroticism and extraversion among adults [27]. Hence, it is important that personality assessment should be handled carefully, since it could be vulnerable to cultural influences and age-specificity [28].

### 1.3. Five-Factor Model of Personality

The Five-Factor Model (FFM) of personality has been influential, since the late 20th century, in recognizing variations in personality traits [29]. Traits refer to the “dimensions of individual differences in tendencies to show consistent patterns of thoughts, feelings, and actions” (p. 25) [15]. These traits are generally known as the five personality traits—neuroticism, extraversion, openness, agreeableness, and conscientiousness [30]. Most scholars have accepted these five personality dimensions. Each trait represents an individual’s distinctive characteristics [31].

A growing body of evidence shows the existence of a strong relationship between these five personality traits and subjective well-being [17,18,19]. Interestingly, recent research suggests that well-being and mental health share common and interrelated predictors [20]. Because of these complex interrelationships, recent studies on mental health and well-being include both depression and life satisfaction as variables in their investigation [22,32].

According to the FFM, personality traits could explain an individual’s self-concept and attitudes toward self [30]. Consistent with this notion, numerous studies [6,8,33] have stated that personality domains are strong predictors of attitudes toward own aging. Therefore, we selected the FFM for investigating the multidimensional relationship between the Big Five personality traits and mental health of older adults. Gerontologists have commonly used the FFM as a framework for a number of studies due to its broader applications [15] and the provision of an integrated descriptive model for research [31].

Emerging research [34] suggests that the mediating function of aging attitudes in the association between personality domains and well-being differs across age and cultural contexts. In this respect, further research remains to be done in the African context, such as in Ethiopia, which is less likely influenced by Western culture [35]. Park and Hess [34] also stressed on the necessity of including a five-factor personality measure that encompasses more than three items per trait to be used for further investigation. Hence, this study aimed to investigate whether attitude toward aging mediates the relationship between the Big Five personality traits and mental health, i.e., depression and life satisfaction of elderly individuals.

## 2. Materials and Methods

### 2.1. Participants and Sampling Procedures

A cross-sectional survey was conducted with 438 retired Ethiopians. The participants were selected through multi-stage cluster random sampling from Addis Ababa (the capital city of Ethiopia) dwellers. Out of 10 districts [36], six districts were randomly drawn. Further, twelve pension payment centers were chosen proportionally. Lastly, participants were selected from each center via convenience sampling. Older adults with severe health issues and visual and hearing impairments were excluded from the study. Being between 60 to 69 years old and pension receiver, former public servant, and retired from the former career were used as inclusion criteria.

In total, 494 individuals completed self-report questionnaires. However, during the analysis, 56 respondents were excluded because they did not provide complete information. Consequently, the number of participants reduced to 438 (316 men and 122 women). The age of the participants ranged from 60 to 69 years, with a mean age of 64.81 years (*SD =* 3.22). A detailed description about sample size determination and study procedures were presented in a previous paper [37].

### 2.2. Measures

All the questionnaires were translated to Amharic (local language) from English, and back and forth, with the help of language experts. In addition, a preliminary pilot study was conducted on 50 retired individuals (male = 34, female = 16) prior to the main study. The participants of the pilot study were aged 60 to 69, with *M* = 64.86 and *SD* = 3.69. The pilot test was conducted in June 2018 mainly to test for the face validity and reliability of the instruments.

Based on the results and suggestions provided by participants of the pilot study, minor adjustments were made to some items. For instance, they helped us select a better term (free from negative connotation in the local context) in the local language to be used for ‘old age’ to indicate specific group without prejudice. Regarding to reliability, most of the instruments obtained good reliability (the Cronbach’s alpha for the overall scales: AAQ = 0.85, Mini-IPIP = 0.52, CES-D Scale = 0.71, and SWLS = 0.72) during the pilot study. Finally, the data for the main study were gathered from July to October, 2018.

*Demographic characteristics:* Data regarding participant’s age, gender, marital status, educational level, income, and living arrangement were collected.

*Attitudes to aging questionnaire (AAQ):* The AAQ consists of 24 items with which older adults themselves can express their attitudes toward the process of aging [38]. It includes a three-factor model encompassing psychosocial loss (PSYSOLOSS), physical change (PHYCH), and psychological growth (PSYGRO). Each dimension constitutes eight items with scores ranging from 8 to 40, and each response is rated on a five-point Likert-type scale ranging from *not true at all* (1) to *extremely true* (5). The Cronbach’s alpha for PSYSOLOSS, PHYCH, and PSYGRO were 0.70, 0.73, and 0.59, respectively [39]. Sample items include “As I get older, I find it more difficult to make new friends (PSYSOLOSS)”, “Problems with my physical health do not hold me back from doing what I want (PHYCH)”, and “As people get older, they are better able to cope with life (PSYGRO)”.

The reliability of the overall scale was 0.86 in this study, and of PSYSOLOSS, PHYCH, and PSYGRO were 0.84, 0.82, and 0.88, respectively. The PSYSOLOSS dimension was reverse-scored to match the other subscales. Thus, the total scores of each domain were considered in the analysis, where higher scores represent a positive attitude toward aging [38].

*Personality Factors:* A short form constituting a 20-item Mini-IPIP personality scale, derived from the International Personality Item Pool was utilized to measure the five-factor model of personality [40]. The instrument was developed and validated across five studies, revealing an internal consistency above α = 0.60 on each dimension [41].

Participants were asked to rate their feelings about themselves using a five-point scale ranging from *very inaccurate* (1) to *very accurate* (5). Negatively stated items were reverse-scored, and the average score of each of the five dimensions was included in the analysis. The Cronbach’s alpha for the overall scale was 0.65 in this study, and openness, conscientiousness, extraversion, neuroticism, and agreeableness were 0.68, 0.67, 0.75, 0.72, and 0.66, respectively.

*Depression:* Depression was measured using the short-form of the Center for Epidemiological Studies Depression Scale (CES-D Scale). It is a 10-item scale designed to measure depression symptoms in the general population [42]. The CES-D Scale is widely used by researchers, and is confirmed to have good validity and reliability [43].

The internal consistency of the scale in this study was 0.78. The items of the CES-D Scale assess the symptoms experienced by the respondent in the past week, and are rated on a four-point scale ranging from *rarely or none of the time* (less than 1 day) to *most or all of the time* (5–7 days). The overall score was used for the analysis, and higher scores were taken as indicators of an increased level of depression [44].

*Life Satisfaction:* A 5-item Satisfaction with Life Scale (SWLS) was used to measure life satisfaction [45]. The SWLS has a good reliability (i.e., a Cronbach’s alpha of 0.88), as reported by [46]. The Cronbach’s alpha for SWLS in our study was 0.76. Participants were asked to indicate the extent to which they agree or disagree with each item on a five-point Likert-type scale, ranging from *strongly disagree* (1) to *strongly agree* (5). The mean score of the items was considered for the analysis, where higher values indicate better satisfaction [45].

### 2.3. Statistical Analysis

The data were analyzed using IBM SPSS 21.0 (IBM, Armonk, NY, USA) statistical software. Descriptive statistics, bivariate correlation, mean, and standard deviation were calculated to summarize demographic characteristics and study variables. The Bonferroni correction was used to evaluate the significance of the correlation coefficients among the study variables. Accordingly, a corresponding *p*-value of <0.006 was considered to be statistically significant. Multiple regression analysis was performed to determine the relationships among the predictor, mediator, and criterion variables. Path analyses were conducted using Amos to investigate the relationship between personality factors and mental health and possible mediators, i.e., attitude to aging subscales. We conducted the path analyses using a 95% confidence interval (CI) with 5000 bootstrapped samples to measure the significance of the mediation effect. A bias-corrected bootstrap CI estimation was performed to detect the nonzero effect [47].

## 3. Results

As shown in Table 1, most of the participants were male (72%), married (71%), and had attained college and above education (56%). Nearly 90% of the respondents were living with their spouse and/or others, whereas the rest lived alone. Seventy-eight percent of the participants indicated that their monthly income was 3000.00 ETB (Ethiopian *birr*, equivalent to 300 USD at the time of data collection) and/or below.

A regression-based path analysis was conducted to determine whether AAQ subscales mediate the relationship between the Big Five personality factors and mental health, measured in terms of depression and life satisfaction. Prior to running the analysis, the correlations among the predictor variables were conducted, and PSYGRO and PHYCH had a high correlation (r = 0.55). Besides, a regression analysis was conducted to check how this collinearity affects the tolerance values. We found that all tolerance values were well over (1-*R*^2^ = 0.87), except for PSYGRO and PHYCH (tolerance = 0.68 and 0.65, respectively). Finally, the problem was solved by aggregating PSYGRO and PHYCH and named PSYGRO_PHYCH. The PSYGRO_PHYCH as an aggregated variable measures an individual’s attitude toward physical change due to aging and psychological growth. In addition, scatterplots were visually inspected and found that the predictors and criterion variables related in a linear way. The values of skewness and kurtosis in Table 2 illustrated that the distribution of all variables were approximately normal. Overall, the data satisfied the assumptions of linearity, normality, and multicollinearity.

The results of Pearson’s correlation analysis of the study variables are presented in Table 2. Accordingly, depression exhibited a significant negative correlation with PSYSOLOSS (r = −0.33, *p* < 0.001) and PSYGRO_PHYCH (r = −0.39, *p* < 0.001). However, life satisfaction had a significant positive correlation with PSYSOLOSS (r = 0.24, *p* < 0.001), but not with PSYGRO_PHYCH. Except for extraversion, all personality factors were significantly associated with depression. Moreover, agreeableness, openness, and neuroticism were significantly correlated with life satisfaction. Whereas, extraversion and conscientiousness were not found to be significantly related to life satisfaction.

Furthermore, additional correlation analysis between personality factors and demographic characteristics was performed. The results suggested that the participants with college and above education reported lower scores on openness, neuroticism, and extraversion (r = −0.21, *p* < 0.001; r = −0.12, *p* < 0.05; r = −0.10, *p* < 0.05, respectively), and higher scores on agreeableness (r = 0.12, *p* < 0.05). However, the correlation between the personality traits and age and gender was not statistically significant.

The following indices (Table 3) were computed to evaluate the goodness of fit of the models to the data: chi-square statistic (χ^2^), χ^2^/df ratio, Normed Fit Index (NFI), Tucker-Lewis Index (TLI), Comparative Fit Index (CFI), Standardized Root Mean Square Residual (SRMSR), and Root Mean Square Error of Approximation (RMSEA). The overall goodness of fit statistics showed that the models fit the data well [48].

**Figure 1:**
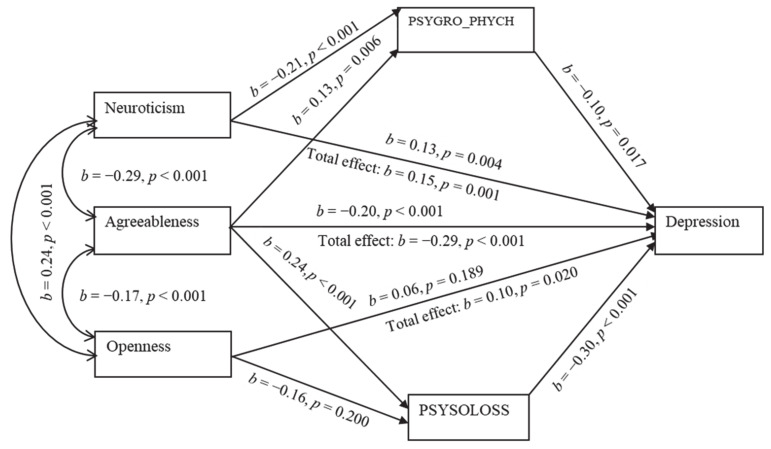
Mediation of AAQ subscales in the relationship between personality and depression.

The results of the mediation path analysis for the depression model (Figure 1) revealed that the predictor variables together estimated 25% of the variance (*p* < 0.001). Specifically, the relationship between neuroticism and depression was partially mediated by PSYGRO_PHYCH (β = 0.02, *p* = 0.009). In addition, the direct path from neuroticism to depression was still significant (β = 0.13, *p* = 0.004). The total direct and indirect effects were reported significant (β = 0.15, *p* = 0.001). Therefore, a high score on neuroticism would negatively correlate with the attitude toward aging, which might lead to depression. The mediating test of indirect effect showed that PSYSOLOSS and PSYGRO_PHYCH jointly mediated the relationship between agreeableness and depression (β = −0.09, *p <* 0.001). A significant negative direct association exists between agreeableness and depression (β = −0.20, *p <* 0.001). Individuals with high scores on agreeableness reported a considerably better attitude toward aging and a lower tendency toward depression.

Conversely, there was no significant direct relationship that existed between openness and depression apart from an indirect (β = 0.05, *p* = 0.001) one. Therefore, the association between openness and depression is most likely to be mediated by PSYSOLOSS. Openness to experiences in this particular case seems to have been negatively related to the self-perception of aging, which might have resulted in depression. In short, agreeableness and openness exhibited a significant total effect on mental distress (β = −0.29, *p* < 0.001 and β = 0.10, *p* < 0.020, respectively).

**Figure 2:**
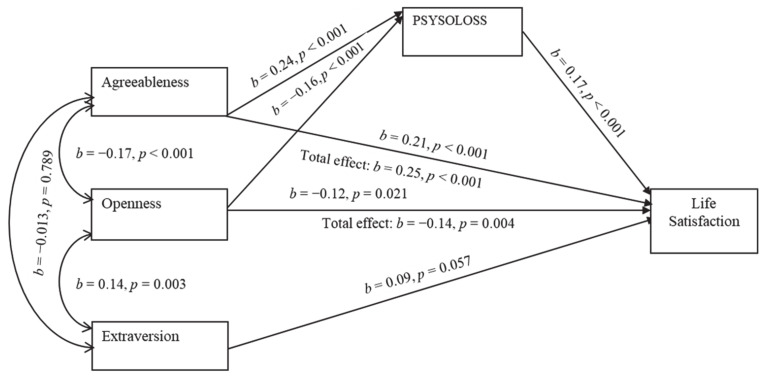
Mediation of PSYSOLOSS in the relationship between personality and life satisfaction.

The second path analysis (Table 4 and Figure 2) was conducted to assess the mediation of PSYSOLOSS in the relationship between personality and life satisfaction. The results illustrate (Table 5) that openness and agreeableness had a significant total effect on life satisfaction (β = −0.14, *p* = 0.004 and β = 0.25, *p* < 0.001, respectively). These associations were partially mediated by PSYSOLOSS. Individually, PSYSOLOSS mediated the relationship between agreeableness and life satisfaction (β = 0.04, *p* < 0.001), as well as the association between openness and life satisfaction (β = −0.03, *p* = 0.001). In addition, life satisfaction significantly and directly correlated to agreeableness (β = 0.21, *p* < 0.001) and openness to experience (β = −0.12, *p* = 0.02).

Therefore, agreeableness appears to have a positive relationship with attitude toward aging (PSYSOLOSS) and life satisfaction, which confirmed the results of correlational analysis in the above Table 2. Conversely, openness to experiences negatively related to an individual’s life satisfaction directly, and a person’s attitude toward one’s own aging. Even if it appeared as a significant predictor of life satisfaction in a hierarchical regression model, extraversion lost its significance during the path analysis. In addition, it was not found to be a statistically significant predictor of both life satisfaction and depression. Conscientiousness was also excluded in the beginning because of its inability to contribute to either of the models.

## 4. Discussion

Attitude about aging experiences is likely to explain an individual’s daily actions and mental health. The results of correlation analysis depicted that more positive views toward self-aging were negatively associated with depression and positively correlated to better life satisfaction. These findings are in line with previous studies that found a strong relationship between better feelings about aging and with low depression [8] and improved life satisfaction [12]. On the other hand, negative attitudes toward aging was also positively linked with increased aging. This finding was consistent with that of Loi et al. [8], who argue that being younger is associated with better attitudes toward aging.

The aim of the present study was to explore the mediating role of attitude toward aging in the relationship between the Big Five personality traits and mental health. Our findings show that attitude toward aging plays a significant role in explaining the relationship between personality domains and mental health among older adults. As the recent evidence [4,5] suggests, we found that neuroticism and openness to experience were positively associated with depression, while agreeableness was negatively associated. Although our results confirmed the previously claimed [3,5] positive correlation between neuroticism and depression, our results further state that the relationship is also partially mediated by attitude toward aging. Supporting this new result is a study by O’Shea et al. [4] indicating that individuals with high scores on neuroticism tend to feel depressed and negatively perceive their aging, which could further exacerbate the symptoms of depression.

Agreeableness, on the contrary, was found to be negatively associated with depressive symptoms, which supports the findings of previous studies [3,23]. This result should be carefully interpreted, as the relationship between agreeableness and depression was partially facilitated through attitudes toward aging. The more people express their opinions and managed relationships, the less likely they are to be depressed and of having a better perception of their aging. Although openness to experience is positively linked to depression [3,5], our study suggests that the association between the two variables is likely to be mediated by PSYSOLOSS. However, the direct relationship between openness and depression was not statistically significant in this study.

Conversely, we also measured satisfaction with life as a crucial indicator of the positive outcome of mental health. The results indicated that participants with high scores on agreeableness reported better life satisfaction than their counterparts. This finding partially agrees with the previous study by Volodinaa et al. [26], who reported a positive relationship between agreeableness and life satisfaction. However, our results disagree with their finding, as neuroticism and extraversion dimensions were not found to be significantly linked to life satisfaction. This may be due to cultural variation, which may have a strong influence on personality [27].

Moreover, contrary to the findings of Volodinaa et al. [26], we found that openness to experience was negatively correlated with life satisfaction. This inconsistency may be due to cultural variation, especially as openness to experience for Africans (Ethiopians) appears to differ from that of the Western world [34].

### 4.1. Theoretical Considerations and Practical Implications

The FFM employed in this study helped us to explain the association between personality factors and mental health, as has been previously claimed [8,30]. Nevertheless, the partial mediation of attitudes toward aging needs to be considered. The strength of such mediations depends on specific personality domains as well as their relationships to positive (life satisfaction) and negative (depression) outcomes of mental health.

Our results reveal the mediating role of attitude toward aging among retired elderly people for future research. However, the mediating function of attitudes toward aging in the association between personality factors and mental health may vary due to age and cultural contexts [34]. Therefore, personality traits, attitudes toward aging, and cultural context are important while designing interventions for improving mental health among older adults.

### 4.2. Limitations and Future Directions

The results of this study should be interpreted with some limitations. Despite the Big Five personality domains analyzed in this study [41], the issue of how each personality facet would predict depression and life satisfaction was not fully addressed. Thus, further research is recommended to identify the mediating role of attitude toward aging on the relationship between each personality facet and depression and life satisfaction. In addition, given the age limit (i.e., 60 to 69) of our participants, the interpretation of the developmental aspect of the five-factor personality [16] on attitudes toward aging might not be possible. Therefore, we recommend the inclusion of samples with a broader age range for further study. Finally, the present study revealed that attitude toward aging mediated the relationship between personality and mental health among senior individuals. Hence, it would be difficult to provide a causal inference from the findings of this study. We highly recommend further investigations on this area to explore the causal relationship among these variables by employing longitudinal study design, which covers the limitations related to the cross-sectional design.

## 5. Conclusions

This study provides evidence supporting the mediating effect of attitude toward aging (PSYSOLOSS and PSYGRO_PHYCH) in the relationship between three personality traits, namely neuroticism, agreeableness, and openness, and depression. Higher depressive symptoms reported a significant direct association with high scores on neuroticism and low scores on agreeableness. Conversely, agreeableness and openness reported significant positive and negative associations with life satisfaction, respectively, and attitude toward aging (PSYSOLOSS) explained these relationships. Therefore, regardless of circumstances, personality could influence life adjustment among older adults.

## Figures and Tables

**Table 1:** Participants’ Characteristics.

Variables	Total
	*n* (%)
* Age (years)	
60–69	64.81 (3.32)
Gender	
Male	316 (72.10)
Females	122 (27.90)
Marital status	
Married	312 (71.20)
Divorced/widowed/single	126 (28.80)
Living arrangement	
With spouse or/and others	389 (88.80)
Live alone	49 (11.20)
Education	
≥College	243 (55.50)
≤High school	195 (44.50)
Income	
>3000.00 ETB	96 (21.90)
≤3000.00 ETB	342 (78.10)

Note. * The mean was used along with the standard deviation in parentheses.

**Table 2:** Mean, standard deviation, and correlations among the study variables.

Variables	Skewness	Kurtosis	1.	2.	3.	4.	5.	6.	7.	8.	9.
1. Depression	0.63	−0.27	—								
2. Life satisfaction	−0.13	−0.84	−0.33 *	—							
3. PSYSOLOSS	−0.94	0.15	−0.39 *	0.24 *	—						
4. PSYGRO_PHYCH	−0.78	0.58	−0.22 *	0.05	0.12	—					
5. Conscientiousness	0.35	−0.22	0.19 *	−0.13	−0.21 *	−0.26 *	—				
6. Extraversion	−0.41	−0.77	0.10	0.06	−0.05	−0.06	−0.11	—			
7. Neuroticism	0.95	0.69	0.25 *	−0.14 *	−0.11	−0.25 *	−0.31 *	0.18 *	—		
8. Agreeableness	−0.43	−0.33	−0.35 *	0.27 *	0.26 *	0.19 *	−0.21 *	−0.01	−0.29 *	—	
9. Openness	−0.02	−0.65	0.20 *	−0.17 *	−0.20 *	−0.16 *	0.26 *	0.14 *	0.24 *	−0.17 *	—
Mean			9.60	16.42	32.52	27.79	2.67	3.34	2.22	3.57	2.99
Standard deviation			5.90	5.13	6.87	6.55	0.81	0.88	0.81	0.81	0.86

Note. * *p* < 0.006 (Bonferroni corrected *p*-value).

**Table 3:** The overall goodness of fit statistics for the models.

Models	CMIN/df	NFI	TLI	CFI	RMSEA	SRMSR
Model 1 (Figure 1)	1.539	0.983	0.969	0.994	0.035	0.0228
Model 2 (Figure 2)	0.209	0.998	1.071	1.000	0.000	0.0054

Note. Estimation, standard error, and CI were conducted on 95% confidence interval bootstrapping prediction.

**Table 4:** Direct and indirect effects of PSYGRO_PHYCH and PSYSOLOSS on the relationship between personality and mental health.

Model Pathways	Β	Boots SE	95% CI	*p*-Value
Model 1: Direct pathway				
Neu → Dep	0.125	0.043	[0.040, 0.208]	0.004
Agr → Dep	−0.202	0.044	[−0.285, −0.112]	0.000
Ope → Dep	−0.202	0.044	[−0.285, −112]	0.189
Model 1: Indirect pathway				
Agr → PSYGRO_PHYCH and PSYSOLOSS → Dep	−0.086	0.020	[−0.132, −0.051]	0.000
Neu → PSYGRO_PHYCH → Dep	0.022	0.010	[0.005, 0.047]	0.009
Ope → PSYSOLOSS → Dep	0.049	0.016	[0.020, 0.084]	0.001
Model 2: Direct pathway				
Agr → SWL	0.206	0.049	[0.110, 0.297]	0.000
Ope → SWL	−0.116	0.049	[−0.213, −0.019]	0.021
Ext → SWL	0.091	0.046	[−0.002, 0.178]	0.057
Model 2: Indirect pathway				
Agr → PSYSOLOSS → SWL	0.040	0.015	[0.016, 0.075]	0.000
Ope → PSYSOLOSS → SWL	−0.027	0.011	[−0.056, −0.010]	0.001

Note. Estimation, standard error, and CI were conducted on 95% confidence interval bootstrapping prediction. Neu = Neuroticism, Agr = Agreeableness, Ope = Openness, Ext = Extraversion, Dep = Depression, SWL = Satisfaction with life.

**Table 5:** Total effects of the models on mental health (depression and life satisfaction).

Models	Β	Boots SE	95% CI	*p*-Value
Model 1: Depression				
Neuroticism	0.147	0.042	[0.061, 0.226]	0.001
Agreeableness	−0.288	0.042	[−0.366, −0.201]	0
Openness	0.104	0.044	[0.016, 0.188]	0.02
PSYSOLOSS	−0.304	0.044	[−0.389, −0.216]	0
PSYGRO_PHYCH	−0.103	0.044	[−0.191, −0.019]	0.017
Model 2: Satisfaction with life				
Agreeableness	0.246	0.046	[0.156, 0.333]	0
Openness	−0.143	0.05	[−0.241, −0.044]	0.004
Extraversion	0.091	0.046	[−0.002, 0.178]	0.057
PSYSOLOSS	0.169	0.048	[0.072, 0.265]	0

Note. Estimation, standard error, and CI were conducted on 95% confidence interval bootstrapping prediction.

## Data Availability

Not applicable.
